# Phosphaturic mesenchymal tumors: radiological aspects and suggested imaging pathway

**DOI:** 10.1007/s11547-021-01412-1

**Published:** 2021-08-28

**Authors:** Mohsin A. M. Hussein, Francesco Pio Cafarelli, Maria Teresa Paparella, Winston J. Rennie, Giuseppe Guglielmi

**Affiliations:** 1 Leicester Royal Infirmary, Infirmary Square, Leicester, LE1 5WW UK; 2 Department of Clinical and Experimental Medicine, Foggia University School of Medicine, Viale L. Pinto, 1, 71121 Foggia, Italy

**Keywords:** Phosphaturic mesenchymal tumors, Oncogenic osteomalacia, Tumor-induced osteomalacia, Fibroblast growth factor 23

## Abstract

Phosphaturic mesenchymal tumors (PMTs) are rare mesenchymal neoplasms of soft tissue or bone origin that can give rise to a challenge in diagnostic imaging. These tumors are frequently associated with tumor-induced osteomalacia, also called oncogenic osteomalacia, which is a rare paraneoplastic syndrome characterized by ectopic secretion of fibroblast growth factor 23, a hormone that regulates serum phosphate level. PMTs show polymorphic features on both radiological findings and histological examination, causing problems in diagnosis owing to their similarity with other mesenchymal tumors. Thus, this paper aims to describe radiological aspects of PMTs and suggest an imaging pathway for accurate diagnosis throughout the evidence from the literature review.

## Introduction

Phosphaturic mesenchymal tumors (PMTs) are rare mesenchymal neoplasms of soft tissue or bone origin that can give rise to a challenge in diagnostic imaging. These tumors are frequently associated with tumor-induced osteomalacia (TIO), also called oncogenic osteomalacia, which is a rare paraneoplastic syndrome characterized by ectopic secretion of fibroblast growth factor 23 (FGF-23). FGF-23 regulates serum phosphate level reducing its renal reabsorption [1] and causes 1,25-di-hydroxyvitamin D3 deficiency with subsequent decreased intestinal phosphate and calcium absorption [2]. That leads to hypophosphatemia, rickets, osteomalacia, and secondary hyperparathyroidism [3]. The first case of TIO was described in 1955 by McCance et al. in a 17-year-old girl who developed osteomalacia and hypophosphatemia. She was found to have a neoplasm in her right distal femur [4], but although her symptoms resolved following excision, initially these were not attributed to the tumor. A few years later, in 1959 Prader et al. stated this association [4, 5]. Since then, over 300 case reports have been reported [6, 7]. In 1972, Evans and Azzopardi observed that TIO-associated mesenchymal tumors were morphologically unique entities [8] and finally in 1987, the term “PMT” was coined to describe these neoplasms. In 2004, Folpe et al. reviewed over 120 cases and found that the majority of cases of TIO were caused by PMTs [9]. PMTs show polymorphic features on both radiological findings and histological examination, causing problems in diagnosis owing to their similarity with other mesenchymal tumors. Current knowledge of the imaging features of PMTs comes largely from case reports and small case series in pathology, endocrinology, and nuclear medicine literature. Many reports are describing pathological and functional imaging characteristics PMTs, but few reports exist on the imaging features using computed tomography (CT) and magnetic resonance imaging (MRI), plain radiographs, and ultrasonographic features. Thus, this paper aims to describe radiological aspects of PMTs and suggest an imaging pathway for accurate diagnosis throughout the evidence from the literature review.

## Epidemiological and clinical features

The vast majority of PMTs occur in middle-aged adults and are small and undetectable on physical examination [10]. They affect both genders equally and can also be seen in the pediatric population and later in life [11, 12]. These neoplasms represent most commonly a benign, disease but nine malignant variants have been described which make up < 2.5% of all reported cases [9, 13]. As far their localizations, in the soft tissues they often involve the extremities, whereas bone tumors commonly occur in the appendicular skeleton, cranial bones, and paranasal sinuses [14]. Adult patients may complain of bone and muscle pain, malaise, and generalized stiffness [9, 15]. These symptoms are typically the result of chronic hypophosphatemia [16]. PMTs represent a rare cause of osteomalacia [17] which is a metabolic disorder of mature bone in which there is defective osteoid mineralization as a consequence of vitamin D deficiency, vitamin D resistance, or hypophosphatemia, due to the secretion of FGF-23. As the bone disease progresses, fractures can occur, affecting dramatically patients’ mobility and significantly disable them. In children, this condition may manifest as rickets which is characterized by muscle hypotonia, delayed development, and bony developmental malformations such as bowing of long bones [4, 5]. Due to the non-specific symptoms of PMTs, diagnosis is often delayed with an average disease course of 6.7 years [18].

## Radiological features

### Plain radiograph

**Fig. 1:**
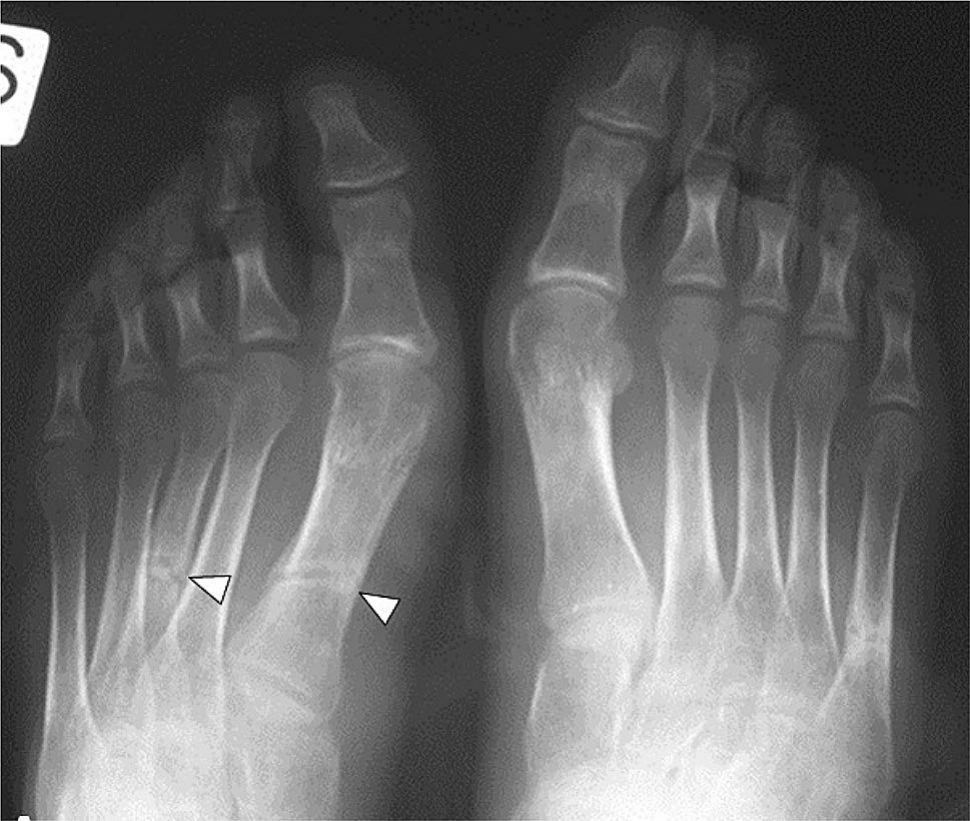
22-year-old man with a rhinopharyngeal vault PMT who presented with a 4-year history of generalized pain and progressive weakness confining him to a wheelchair. Plain radiograph demonstrates multiple insufficiency fractures (arrowheads) with features of osteomalacia in the feet

**Fig. 2:**
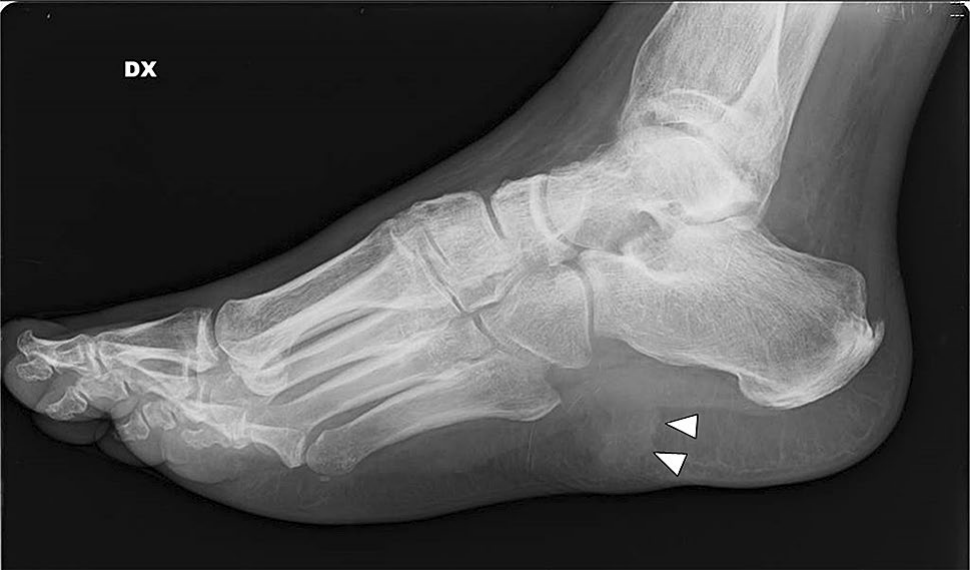
44-year-old man with a PMT of the foot. Plain radiograph demonstrates a faint increased density in keeping with soft tissue calcification on the plantar aspect of the foot

**Fig. 3:**
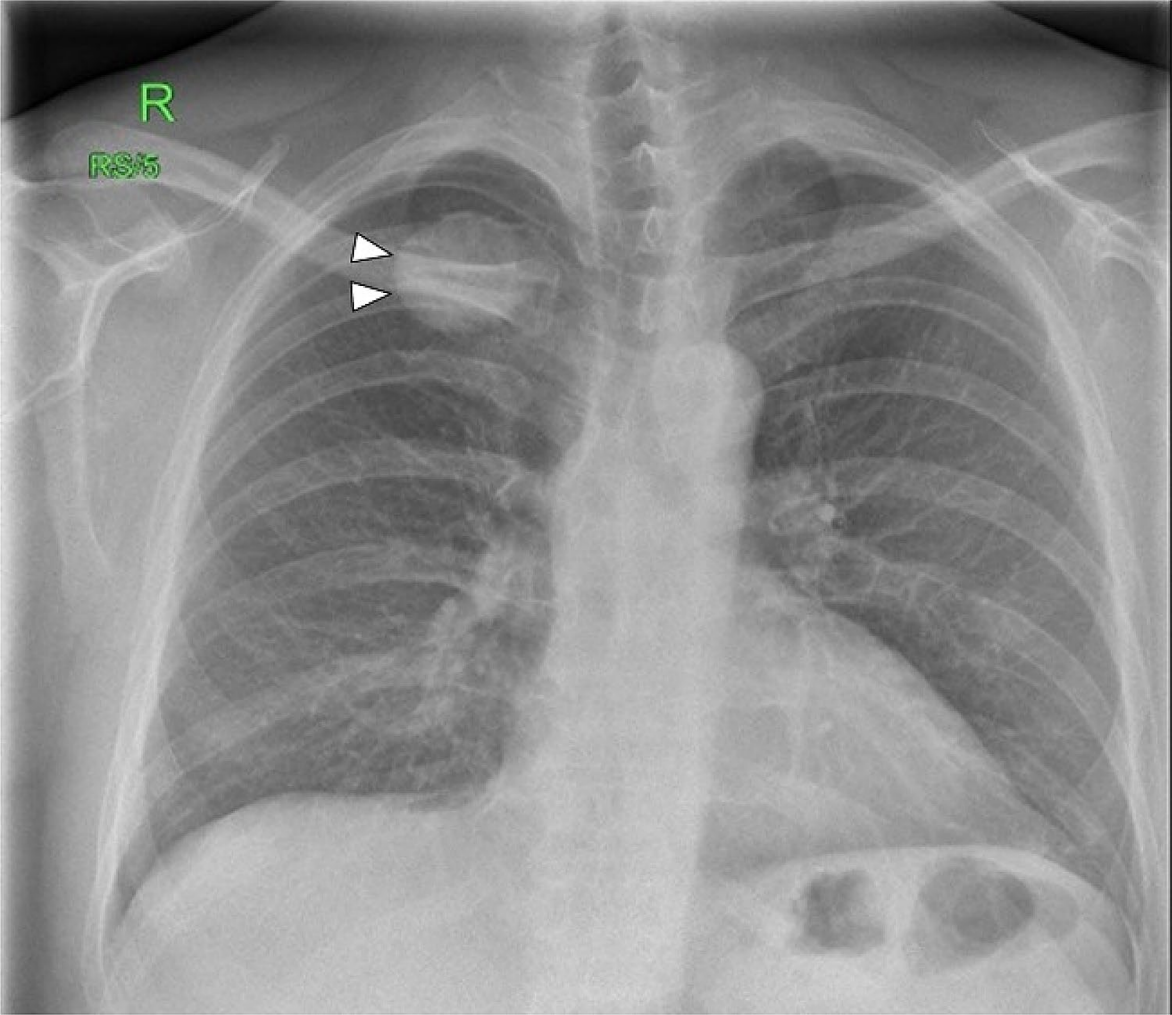
47-year-old man with a PMT of the 4th rib. He presented to the rheumatology service, with an 18-month history of recurrent back pain and unexpected weight loss. On physical examination, he had reduced spinal movement and point tenderness over his sacroiliac joints. Chest X-ray shows a well-defined rounded dense lesion in the 4th rib, with uniform calcification

**Fig. 4:**
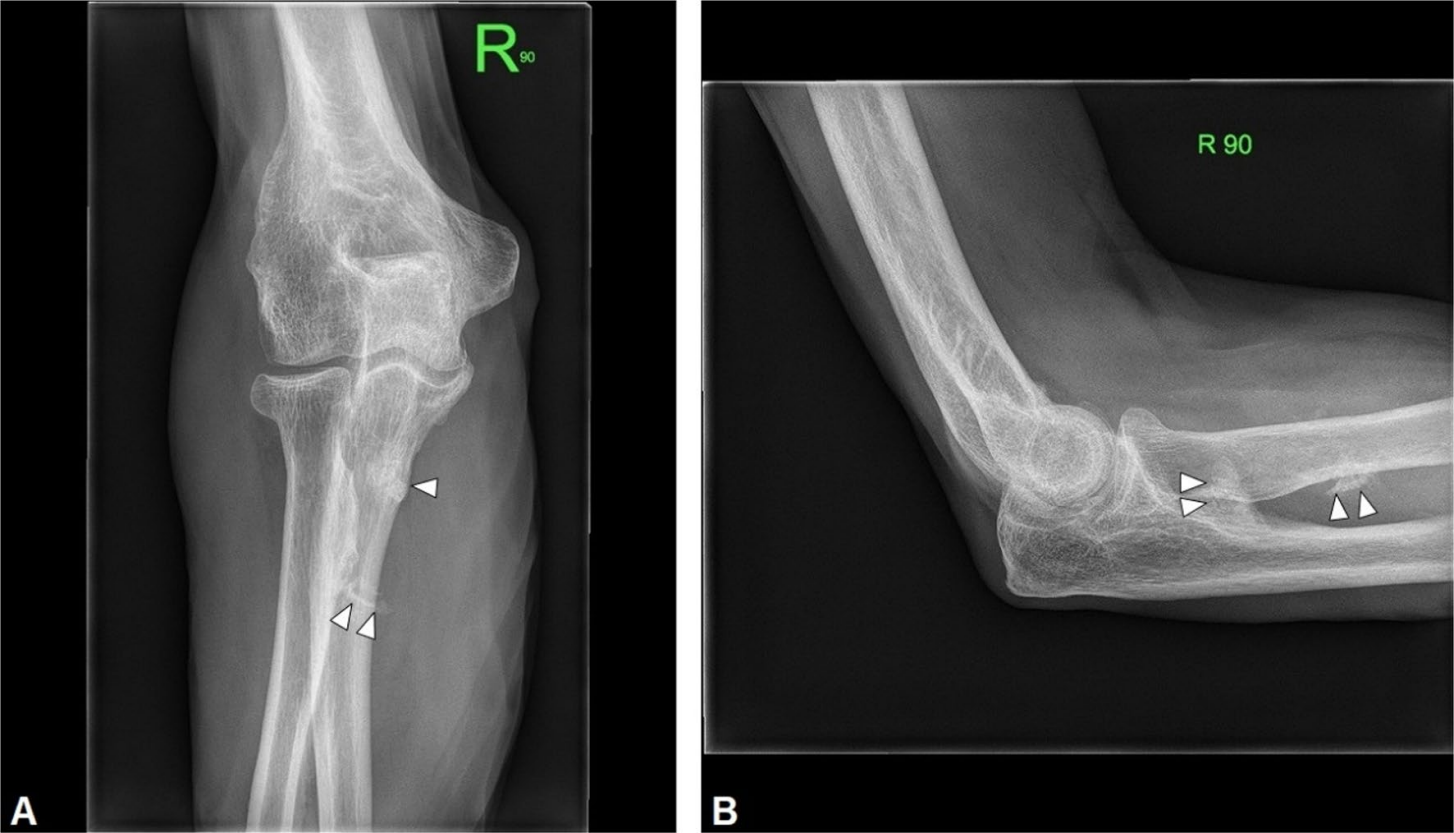
65-year-old man with a PMT of the right elbow. AP (**A**) and lateral (**B**) radiographs of the shoulder demonstrate an ill-defined area of soft tissue calcification in the proximal forearm

X-rays are often the first radiological technique performed in symptomatic patients. Common plain radiographic findings include coarse trabeculae, thinned-out cortex, and Looser’s zones, manifestations of generalized osteopenia in adults, and rickets in children [19]. X-rays of the foot and pelvis commonly demonstrate non-united osteomalacic fractures [18] (Fig. 1). Pseudo-fractures are another feature and may occur more frequently in the femoral neck, knee, pelvis, and ribs; in addition, old fractures are often seen simultaneously [20]. Mineralization of soft tissue has also been reported [21] (Fig. 2). Nevertheless, also PMTs may appear sclerotic and demonstrate calcifications [22] (Fig. 3). In patients with a chronic condition, pelvic deformities and blurring of the pubic symphysis can also be seen [18]. In one case of a malignant PMT variant, chest radiography showed multiple pulmonary nodules with ground-glass appearance [13]. Since these tumors are generally small, they can be difficult to locate on radiographs alone, so further anatomical or functional imaging is usually required [23] (Fig. 4).

### Ultrasound

**Fig. 5:**
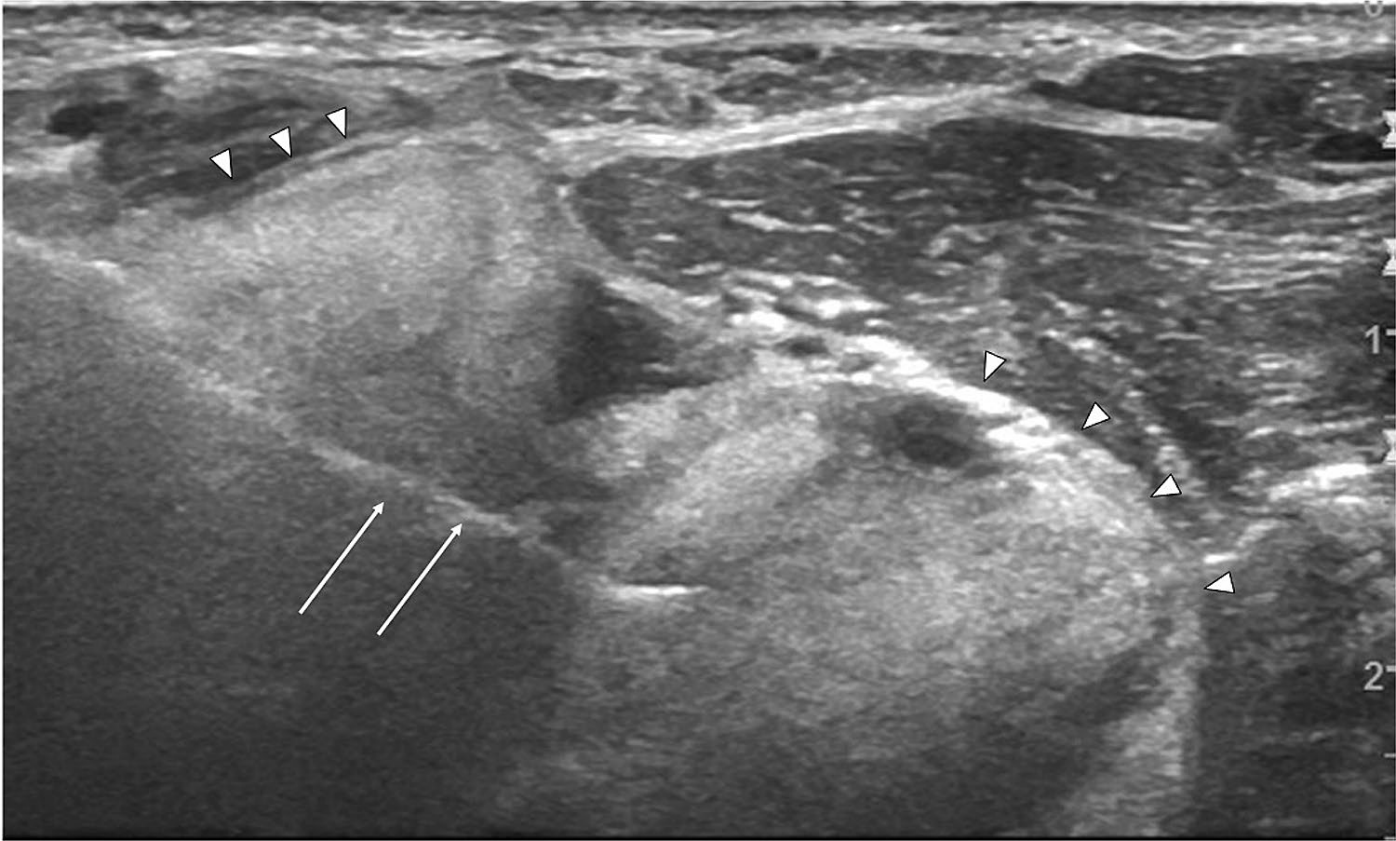
65-year-old man with a PMT of the right elbow. Ultrasound of the proximal forearm shows a large extraosseous component (arrow heads) which was not apparent on plain radiograph. Biopsy needle (arrows)

Ultrasonographic characteristics of PMTs were described by Busquet et al. in a mass located in the right thigh which presented a heterogeneous and hypoechoic structure with well-circumscribed edges [24]. This lesion was differentiated from a lipoma due to a positive Doppler signal around the tumor. This patient had comprehensive cross-sectional imaging of the chest, abdomen, and pelvis during the preceding five years, but the neoplasm was not diagnosed until the patient’s thigh was clinically examined, an ultrasound examination of the mass was requested, and a subsequent confirmatory biopsy was performed. Another case where an ultrasound scan was useful to identify a large extraosseous component that was not present at the initial radiograph was also present in our imaging records (Fig. 5). These cases demonstrated that ultrasound can be an important tool in characterizing PMTs of the appendicular skeleton, which is more amenable to an ultrasound scan. It also highlighted that clinical examination is mandatory in combination with imaging techniques for the diagnosis of PMTs and other bone or soft tissue masses.

### Computed tomography

**Fig. 6:**
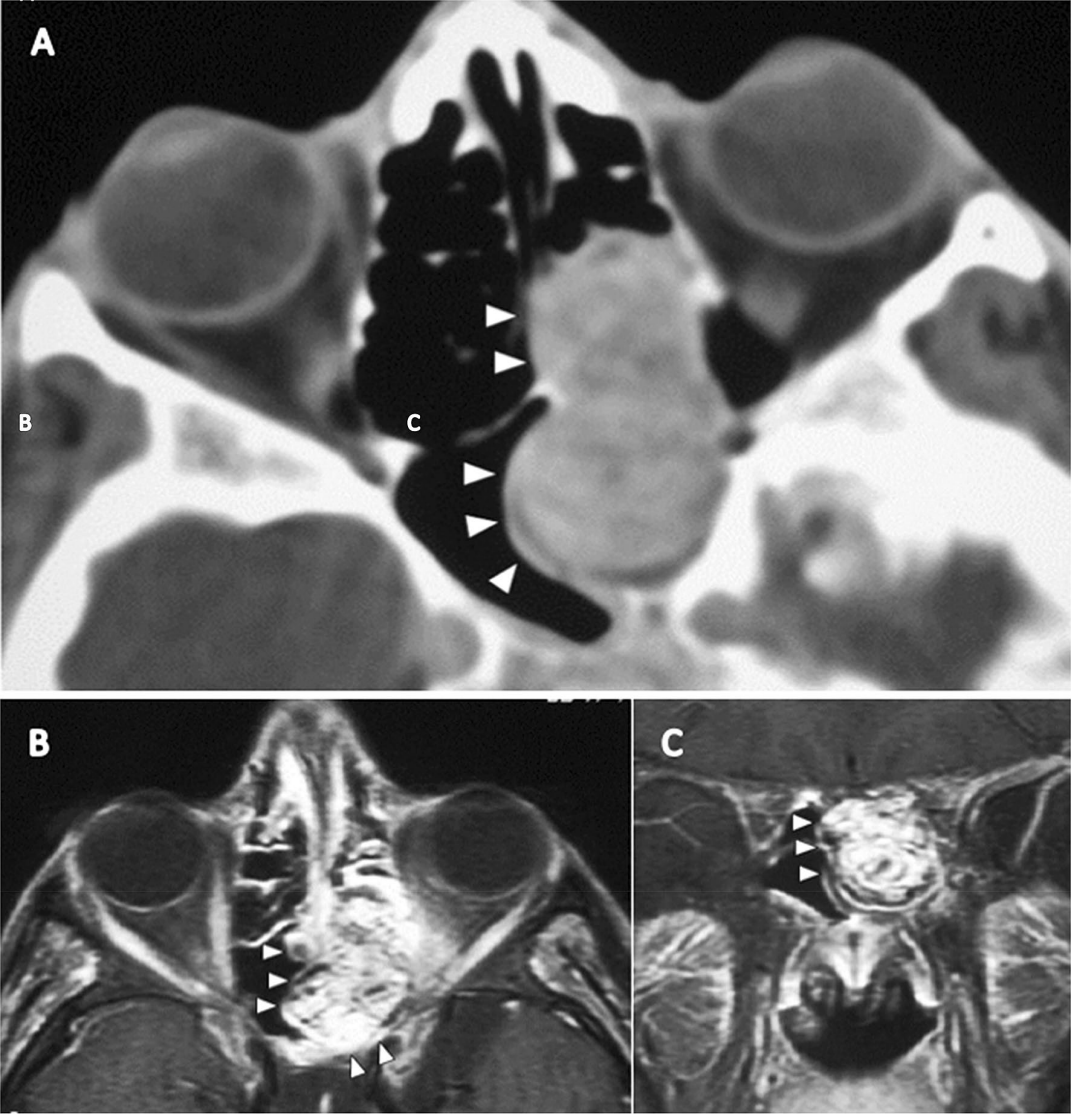
22-year-old man with a rhinopharyngeal vault PMT who presented with a 4-year history of generalized pain and progressive weakness confining him to a wheelchair. (**A**) Axial CT shows an enhancing bulging tumor arising from the left ethmoidal sinus with an extraosseous soft tissue component. Axial (**B**) and coronal (**C**) axial T2 weight spin-echo MR image show a lesion of the left ethmoidal sinus demonstrating an increased signal intensity of the solid mass, with multiple dark foci or vascular flow voids

**Fig. 7:**
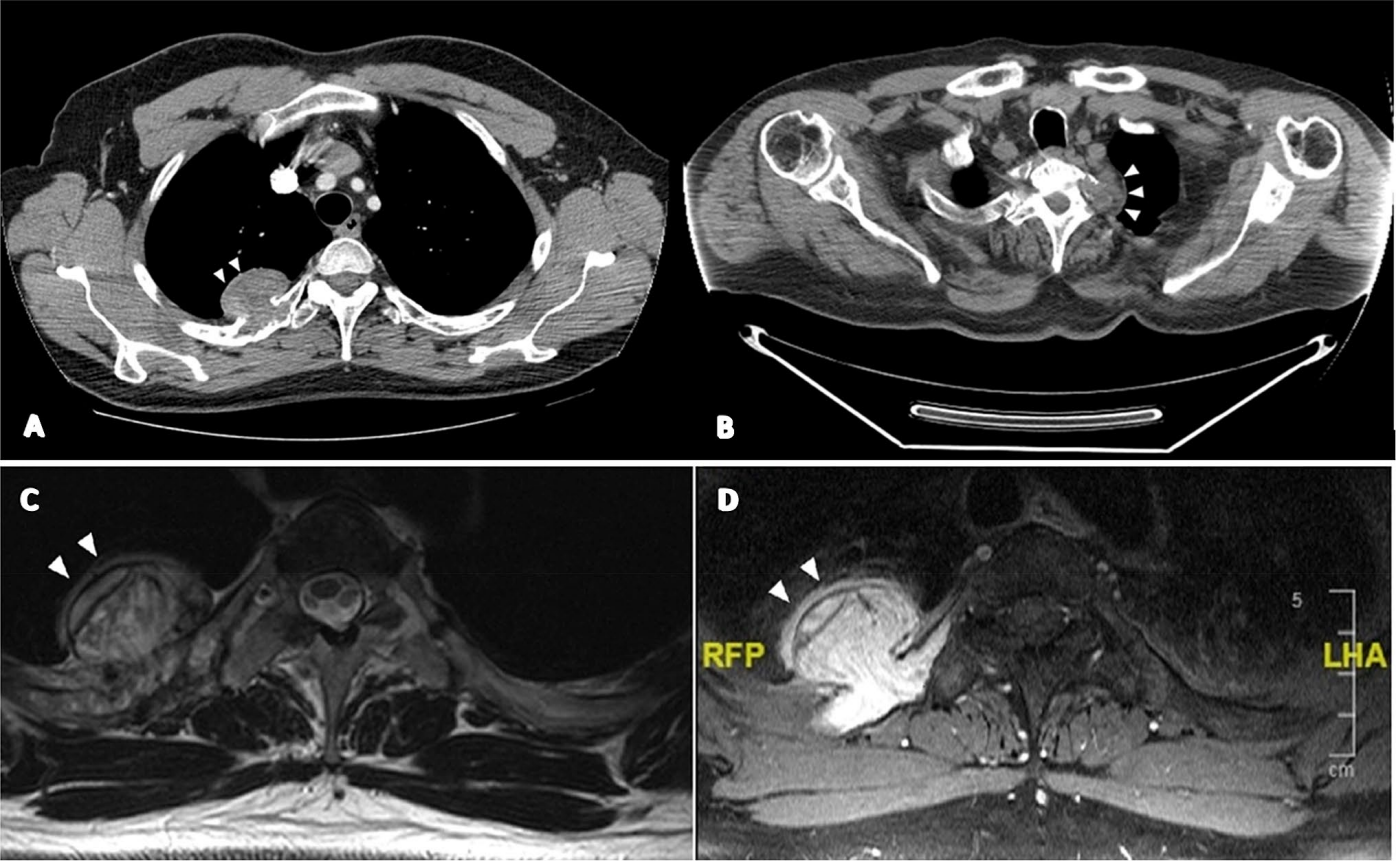
47-year-old man with a PMT of the 4th rib. (**A**, **B**) Axial CT demonstrates a well-defined rounded dense lesion with a mildly calcified internal matrix. (**C**) Axial T2 weighted spin-echo MR image shows a lesion of the 4th rib with mixed hyper- and hypointense signal, well-defined borders, cortical destruction, and extension into the extrapleural fat. (**D**) Axial T1 weighted fat suppressed contrast enhanced image shows intense uniform enhancement of the lesion. The non-enhancing margins (arrowheads) are areas of calcification

Computed tomography (CT) is another imaging technique often used to localize or characterize PMTs. They can easily mimic other pathologies; thus, a high index of suspicion, based on the pattern of fractures, osteomalacia, and characteristics of the mass, should help with diagnosis. PMTs are generally small in size with an average diameter reported as 3.4 cm (1.1–9.8 cm) [23]. Lesions may be located in the soft tissue or bone, with some of the latter showing an extraosseous soft tissue component (Fig. 6A). A key CT finding of PMTs in the presence of an internal matrix (Fig. 7a) may be of punctuated or amorphous and, less commonly, ground-glass appearance [23]. These features probably correlate with the flocculant calcification pattern, which resembles primitive cartilage or bone, observed on histological analysis [25]. Regarding the pattern of osseous involvement, bone lesions are generally osteolytic and less commonly osteosclerotic or mixed osteolytic/osteosclerotic [22] (Fig. 7A, B). A narrow zone of transition can be observed in most lesions [14]. Tumors with a ground-glass appearance and thin sclerotic rims can be confused with fibrous dysplasia [23].

### Magnetic resonance imaging

**Fig. 8:**
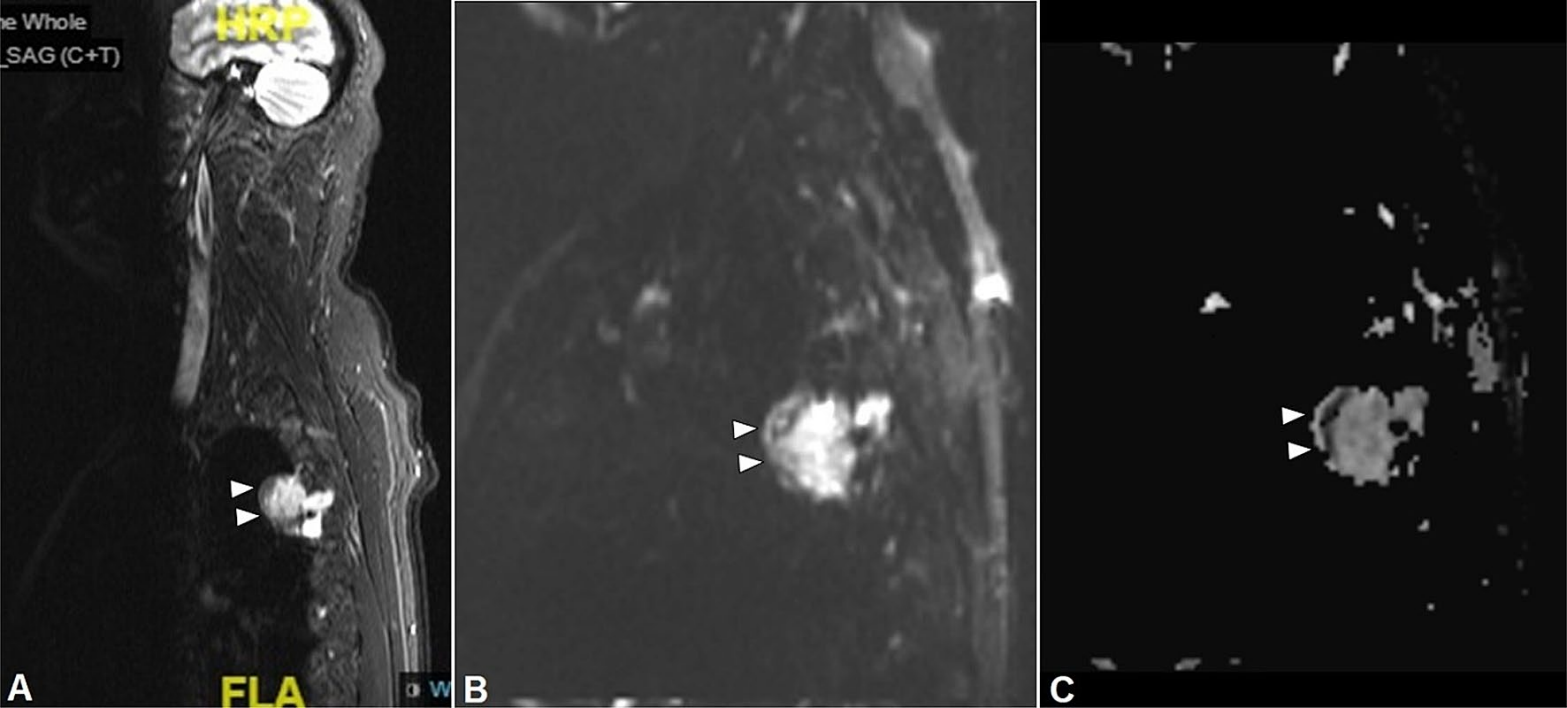
47-year-old man with a PMT of the 4th rib. Para-sagittal STIR sequence (**A**) demonstrates high signal intensity mass with an anterior rim of low signal intensity, arising from the rib. Intense intramedullary edema is noted of the rib. Diffusion-weighted imaging (**B**) with an apparent diffusion coefficient map (**C**) demonstrates restricted diffusion along the anterior margin in keeping with the calcified rim of the PMT

Magnetic resonance imaging (MRI) has been reported to aid in localizing lesions in cases not previously demonstrated on plain radiographs or CT [20, 26]. PMTs show a variety of MRI characteristics according to size and location. Generally, on T1-weighted imaging (T1WI), the lesions predominantly demonstrate an intermediate or hyperintense signal, whereas on T2-weighted imaging (T2WI), the signal is commonly hyperintense and heterogeneous, but intermediate and hypointense signals can also be noted [23, 27] (Fig. 7C, D). A typical feature in larger masses on T2WI increased signal intensity with multiple dark foci or vascular flow voids. This may correlate with the high vascular proliferation of spindled stellate cells, arborizing capillaries, or the hemangiopericytoma-like appearance, found at the histological analysis [22, 25, 28] (Fig. 6B, C). Small tumors demonstrate a homogenous signal on both T1WI and T2WI [29, 30]. After contrast administration, lesions most commonly present a uniform enhancement; nevertheless, some tumors can demonstrate intermediate enhancement and less commonly peripheral enhancement [23]. It has been reported that larger lesions, more frequently than smaller ones, can show a heterogeneous enhancement [28]. PMTs may also reveal an internal fluid–fluid level, internal hemorrhage, and surrounding edematous changes, though these features are non-specific. A more reliable finding would be pathological fractures visible on the same scan as the soft tissue or osseous lesion, in the absence of risk factors or relevant comorbidities, especially in a young patient. To facilitate the localization of PMTs, short tau inversion recovery sequences (STIR) and diffusion-weighted imaging (DWI) can also be performed; both demonstrate high signal intensity for these lesions [20, 21] (Fig. 8A–C). Note should be taken of the poor spatial resolution and T2 shine-through on DWI and careful review of the other sequences taken, before a conclusive diagnosis. In any case, high-resolution STIR sequences with a large field of view, suppressing fat signal and enhancing the signal from tissues with long T1 and T2 relaxation times, such as neoplastic and inflammatory tissue, have been demonstrated to localize lesions [20, 26, 28, 31]. Alternative diagnoses that should be considered for PMTs are hemangioma or fibrosis in soft tissues [21], tenosynovial giant cell tumors in joint or tendon sheaths, fibrous dysplasia in the bone, or even lipomatous tumors [23]; thus, a multi-modality approach to imaging diagnosis is mandatory.

### Functional imaging

**Fig. 9:**
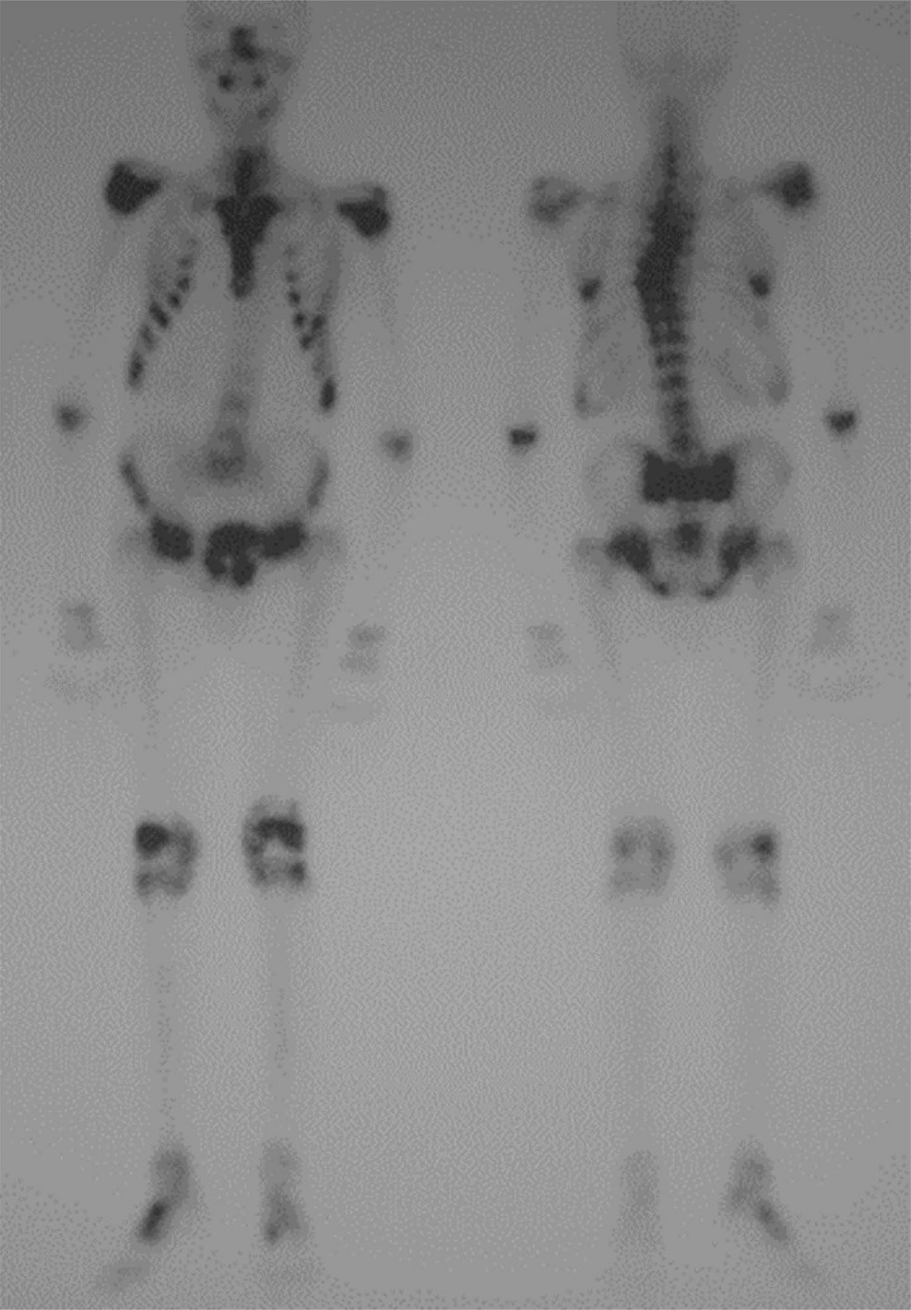
22-year-old man with a rhinopharyngeal vault PMT. Whole-body bone scintigraphy demonstrated multiple areas of increased uptake in the thoracic spine, ribs, pelvis, and limbs with a characteristic H-shape pattern in the sacrum, typically found in insufficiency fractures

PMTs are small, slow-growing tumors that often develop in unorthodox locations such as the mandible, ribs, or sinuses; in these cases, CT and MRI can be non-contributory to their localization; thus, it is necessary to resort to functional diagnosis [32]. In the last two decades, radionuclide imaging demonstrated increasing promise to localize lesions in cases of TIO. PMTs have been shown to express multiple surface receptors with 79% expressing somatostatin [11]; therefore, somatostatin analogs such as 111Inpentettreotide, 99mTc-sestamibi, and 68 Ga-DOTATATE have been used to evaluate cases of TIO [33–37] (Fig. 9). The choice of molecular imaging will depend on the availability at one local institution, with no clear evidence of the superiority of one tracer over the others. In addition, 18F-fluorodeoxyglucose positive emission tomography-computed tomography (18F-FDG PET/CT), which is a useful and commonly available imaging technique, has been used to investigate PMTs. The majority of these lesions appear to be moderately FDG avid with an average SUVmax of 4.1, though they can demonstrate varied metabolic activity (SUVmax 1.5–10.8) [16, 23, 38–40] (Fig. 14). Recent studies have reported only 60% specificity [33, 41] of this technique, in contrast even if early studies reported 99Tc‐HYNIC‐TOC SPECT/CT to show a non-specific radiotracer uptake in 79% of lesions, several recent case series suggested specificity as high as 100% [34, 36, 42, 43]. 111Inpentettreotide has also been used and demonstrated successful radioisotope uptake in between 71 and 95% of cases [35, 37, 39, 41]. Finally, studies of 68 Ga-DOTATATE PET/CT have compared this technique with 18F-FDG PET/CT. Whereas there was an overall delay in the diagnosis of the majority of cases using both techniques, 68 Ga-DOTATATE PET/CT performed better in localizing tumors and reduced the time to diagnosis [42]. In one case, 68 Ga-DOTATATE PET/CT revealed a high uptake lesion which was not demonstrated on 99mTc-sestamibi scintigraphy, 111In-octreotide scintigraphy, and FDG-PET/CT scans [22]. More recently, a case report suggested that 68 Ga-DOTATATE may have increased specificity, due to accurate differentiation between a right inguinal PMT lesion from other lymphoma nodules [44]. Other researchers reported that 68 Ga-DOTATATE was able to localize 100% of lesions compared with FDG PET which localized 50%, supporting the specificity of the technique [33]. These findings can be attributed to the high affinity of 68 Ga-DOTATATE PET/CT for SSTR2, the receptor subtype most frequently expressed on PMTs [45]. As a result, it has been recommended, by many authors, as a first-line functional imaging test in localizing these neoplasms [42, 45, 46]; nevertheless, its use in the diagnosis of PMTs remains off-license. Although many studies and case series have explored molecular imaging techniques, there is no clear consensus on the most appropriate radionuclide scan or its added value when compared with conventional imaging techniques which are more cost-effective and readily available. The reports so far would suggest that 99Tc‐HYNIC‐TOC SPECT/CT and 68 Ga‐DOTATATE PET/CT perform best in identifying a PMT. Due to the variability in size and location of PMTs, whole-body functional imaging would be a recommended technique to effectively localize these tumors before definitive treatment.

## Conclusions and recommended imaging pathway

**Fig. 10:**
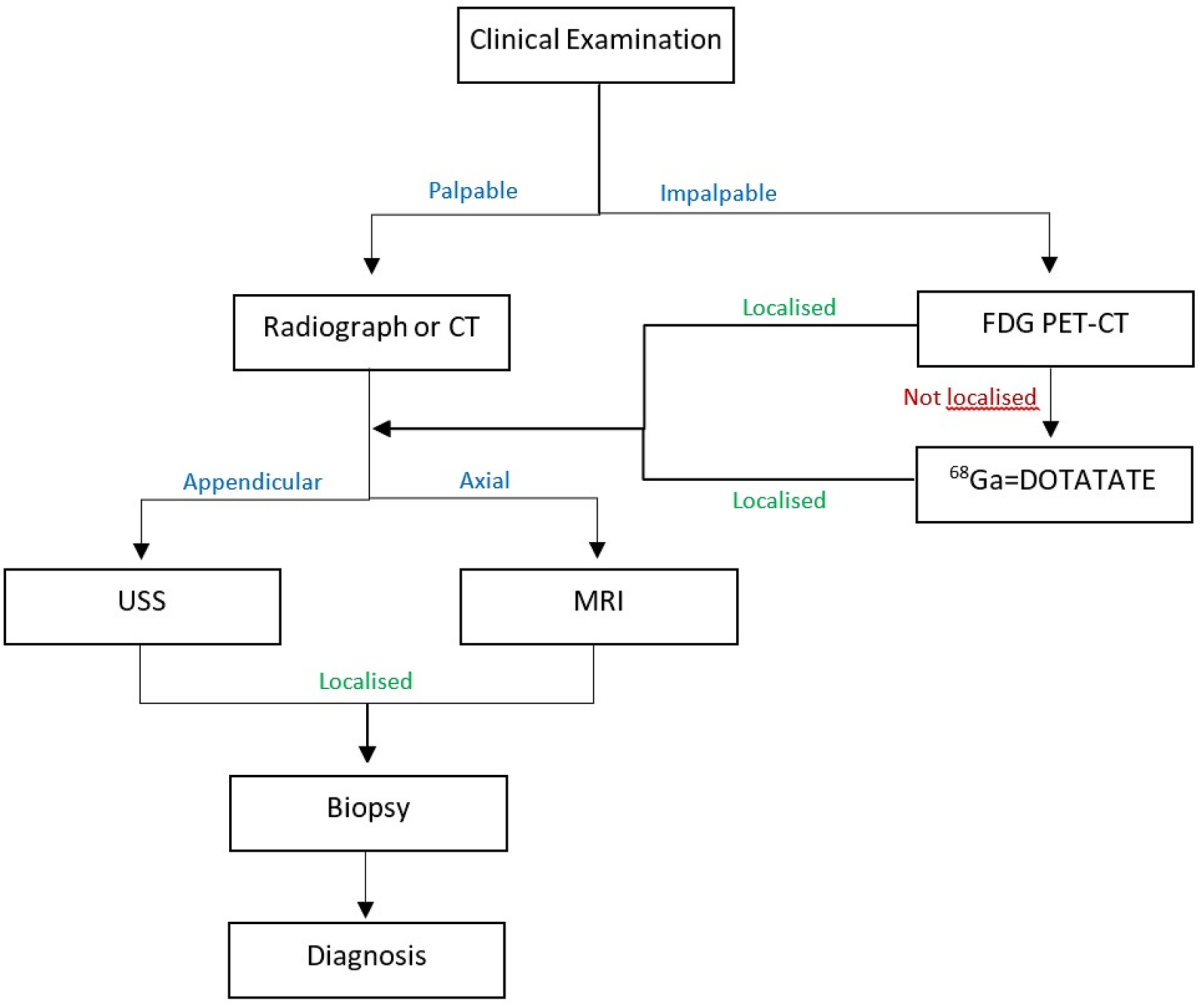
Imaging pathway for PMTs

PMTs remains a relatively rare entity, proving challenging to diagnose because of their heterogeneous morphological aspects on imaging. These can mimic other similar bone or soft tissue neoplasms leading to a delayed diagnosis or in the worst case, a missed diagnosis on imaging. Therefore, throughout the evidence from the literature review, this study aims to suggest an imaging pathway for accurate diagnosis. Firstly, correlation with clinical presentation and laboratory findings is mandatory. Once a lesion is identified clinically and is palpable, an initial radiograph or a CT can be used to localize and classify the lesion, into appendicular and axial skeleton-based neoplasms [47]. Appendicular lesions are amenable to ultrasound examination, characterization, and biopsy. Axial lesions can be characterized by MRI. In impalpable lesions, molecular imaging studies can be utilized to aid in the localization of PMTs and detect impending fractures. 99mTc-sestamibi scintigraphy, 111In-Pentetreotide scintigraphy, 67 Ga-citrate scintigraphy, and 18F-FDG PET/CT can be considered before the off-license use of 68GaDOTATATE PET/CT which can be reserved to evaluate more challenging cases (Fig. 10). Definitive management of the tumor is with surgical resection and postoperative serial blood tests, including serum phosphate FGF-23 level.
